# Stabilization of Styrene Pickering Emulsions Using SiO_2_ Derived from Waste Cement

**DOI:** 10.3390/ma18102281

**Published:** 2025-05-14

**Authors:** Guomei Xu, Jihua Zhang, Defei Long, Huayang Wang, Hanjie Ying, Hongxue Xie

**Affiliations:** 1 Anhui Province Key Laboratory of Conservation and Utilization for Dabie Mountain Special Bio-Resources, School of Materials and Chemical Engineering, West Anhui University, Lu’an 237012, China; 2 State Key Laboratory of Materials-Oriented Chemical Engineering, Nanjing Tech University, Nanjing 210009, China

**Keywords:** Pickering emulsion, SiO_2_, KH550, waste cement, styrene/silica composite microspheres

## Abstract

The initial focus of this study was placed on the conversion of waste into valuable substances. Waste cement was systematically processed to extract silica powder, which was subsequently functionalized with γ-aminopropyl-trimethoxy-silane (KH550) via covalent grafting. The surface-modified silica particles demonstrated optimized amphiphilicity for interfacial stabilization, as confirmed by contact angle measurements. When employed in styrene/water Pickering emulsions, these modified silica particles exhibited exceptional stabilization efficiency, enabling the synthesis of core–shell polystyrene/silica composite microspheres visualized by SEM. It was demonstrated by the results that the Pickering emulsions could be stabilized by SiO_2_ when the appropriate polarity and concentration were achieved. XRD revealed successful silica integration without crystalline phase alteration. Thermogravimetric analysis demonstrated significantly enhanced thermal stability (50.6% residual mass at 800 °C), indicating substantial flame retardancy potential. This waste-to-functional-material strategy not only addresses environmental concerns but also provides an economically viable pathway for advanced polymer composites.

## 1. Introduction

Emulsion polymerization has long been recognized as an important technique in both academic research and industrial applications, particularly for synthesizing polymers, such as rubbers, plastics, adhesives, and functional coatings [1]. Despite their widespread adoption, conventional surfactant-stabilized emulsion systems suffer from inherent limitations that have become increasingly prominent with technological advancements [2]. The critical issues include the residual emulsifiers compromising product purity, the water sensitivity of polymers [3], and the complex post-processing requirements, necessitating urgent improvements to enhance the process efficiency and sustainability [4].

In particular, residual surfactants in the final products can negatively impact their performance, especially in high-purity applications where surfactant removal remains technically difficult. The emergence of Pickering emulsion technology [5], utilizing solid particles as stabilizers, presents a transformative solution by eliminating molecular surfactants while enhancing emulsion stability through interfacial particle control [6]. This approach offers dual advantages: environmentally, it reduces the reliance on toxic low-molecular-weight surfactants [7]; economically, it lowers the production costs [8]. Consequently, Pickering emulsion polymerization—where polymerizable monomers are introduced into particle-stabilized emulsions and polymerized under controlled conditions [9]—has become a central issue in composite material research.

Solid particles used in Pickering emulsions are broadly categorized into the inorganic (e.g., silica [10]) and organic (e.g., starch [11]) types. Among these, silica nanoparticles (SiO_2_) are particularly promising due to their cost-effectiveness, surface modifiability, and optical transparency [12]. Notably, China generates over 200 million tons of construction waste annually, with concrete accounting for a significant portion [13]. Recycling this waste cement could mitigate resource depletion and environmental burdens [14]. In this study, we innovatively adopted waste cement as a raw material for SiO_2_ powder via calcination, addressing both sustainability and waste management challenges.

The efficacy of Pickering emulsions depends on the surface wettability of the stabilizing particles [15]. Optimal stability requires particles with balanced hydrophilic–lipophilic properties. Common strategies to enhance silica’s emulsifying capacity include (1) incorporating auxiliary (co)monomers, (2) the surface modification of silica sols, (3) using alcoholic silica sols, and (4) applying functionalized nanoparticles [16]. Here, we modify waste-cement-derived SiO_2_ with KH550 to improve its hydrophobicity, enabling its application in styrene Pickering emulsions.

Against this backdrop, the primary objectives of this research are twofold. Firstly, to develop a sustainable method for extracting SiO_2_ from waste cement and evaluate its performance as a Pickering emulsion stabilizer. Secondly, to fabricate organic–inorganic composites via Pickering emulsion polymerization using modified SiO_2_, thereby reducing the reliance on raw materials and promoting circular economy principles. By achieving these goals, we aim to bridge the gap between waste management and materials science, offering a scalable solution that meets the requirements of environmental sustainability.

## 2. Materials and Methods

### 2.1. Materials

The waste cement utilized in this study was retrieved from construction waste. The KH550 was procured from Shanghai Macklin Biochemical Technology Co., Ltd. (Shanghai, China). The absolute ethanol (C_2_H_5_OH), methanol (CH_3_OH), sodium hydroxide (NaOH), styrene, azobisisobutyronitrile (AIBN) and other reagents were all sourced from Sinopharm Chemical Reagent Co., Ltd. (Shanghai, China). The styrene monomer (C_8_H_8_) was purified through 5% NaOH aqueous solution and washed to remove the inhibitors. The AIBN was recrystallized from methanol before use. All the chemicals employed in this study were of analytical grade and used as received without additional purification, unless otherwise stated. Deionized water was used throughout this study, and it was prepared in our laboratory using the ZKWL-2001-M ultra-pure water machine (Zhongke Ant Instrument Co., Ltd., Hefei, China).

### 2.2. Extraction of Silica

**Figure 1:**
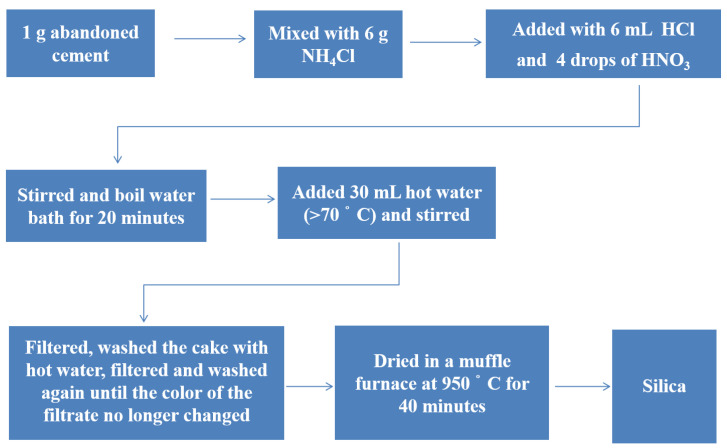
Extraction route for obtaining silica from abandoned cement.

The waste cement was ground into powder using a FW100 plant grinder (Aiyan Biotechnology, Hefei, China) via high-energy shear milling for 10 min. The extraction procedure to obtain silica from the waste cement is illustrated in Figure 1. After undergoing a series of reactions, all the forms of silicon were converted into soluble silicates. Subsequently, they were dissolved in the solution and precipitated in the form of silicic acid hydrogels. The filtration process was carefully executed using a high-quality filter medium to separate the hydrogels from the remaining liquid phase effectively. After filtration, the hydrogels were burned at a precisely controlled temperature (heating rate: 5 °C/min) in a KYS-1200C tubular furnace (Hefei Microelement Laboratory Equipment Co., Ltd., Hefei, China), yielding amorphous silica powder. The key chemical reactions involved in this extraction process are summarized in Equations (1) and (2), which provide a clear insight into the underlying chemical transformations at each stage.

(1) $$SiO_{3}^{2-}+H^{+}\overset{\;acidification\;reaction\;}{\to }H_{2}SiO_{3}$$

(2) $$H_{2}SiO_{3}\underset{\;△\;}{\to }SiO_{2}+H_{2}O$$

### 2.3. Modification of the Prepared Silica

The modification of the prepared silica using KH550 was carried out in the following steps. Firstly, 0.5 g of the prepared SiO_2_ was added to a flask. Subsequently, 10 mL of KH-550 was blended with 100 mL of methanol in a separate container. This mixture was then added dropwise into the flask. The flask was placed in a water bath at 45 °C and underwent continuous stirring for 4 h. Next, centrifugation was performed at 10,000 rpm for 10 min with a high-speed centrifuge (Model HC-3018, Anhui Zhongke Zhongjia Scientific Instrument Co., Ltd., Hefei, China) to separate the dispersed solution. The collected product was then subjected to repeated ultrasonic cleaning using anhydrous ethanol, and this process was repeated three times. The final product was dried under reduced pressure (10 kPa) at 30 °C for 8 h in a DZF-6034 vacuum oven (Yiheng Scientific Instrument Co., Ltd., Shanghai, China). Eventually, the modified SiO_2_ powder was obtained.

### 2.4. Preparation of Pickering Emulsion and Polymerization

A styrene/water Pickering emulsion, stabilized by the modified SiO_2_ retrieved from the discarded cement, was prepared according to the following procedures. First, 0.36 g of the modified SiO_2_ particles and 15 mL of deionized water were introduced into a 50 mL flat-bottomed test tube and then ultrasonically dispersed for 30 min to obtain a homogeneous silica dispersion [17]. Second, 3 mg of AIBN was dissolved in 3 mL of styrene by magnetic stirring at 500 rpm for 15 min at 25 °C to form the oil phase. Subsequently, the oil phase was added to the flat-bottomed test tube. Then, the test tube was placed in an ultrasonic generator and continuously ultrasonicated for 30 min while the temperature was maintained below 30 °C using an ice-water bath for emulsification, finally yielding a uniform styrene/water Pickering emulsion. To investigate the effects of the particle concentration on the emulsion stability, the dosage of the modified SiO_2_ was systematically varied while keeping the other parameters constant. The specific formulations are detailed in Table 1.

The styrene-in-water emulsion polymerization was conducted in a three-necked round-bottom flask (250 mL) equipped with a mechanical stirrer, reflux condenser, and nitrogen inlet. After degassing with N_2_ for 20 min, the system was immersed in a thermostatic oil bath (70 ± 0.5 °C, IKA) for 24 h of polymerization. The resultant mixture was precipitated in 200 mL of anhydrous ethanol, followed by centrifugation (10,000 rpm, 10 min, 25 °C). The collected microspheres underwent three washing cycles with ethanol/water (*v*/*v* = 1:1) and vacuum drying (60 °C, 24 h).

### 2.5. Measurements

An NM910 metallographic microscope (manufactured by Yongxin Optical Co., Ltd. Ningbo, China) was employed to observe the morphology of the silica and styrene/silica composites by applying them to the surface of a glass slide. An M393003 contact angle machine, procured from Shanghai Fang Rui Instrument Co., Ltd. (Shanghai, China), was utilized to test the three-phase contact angle of the prepared silica at the oil–water interface. Specifically, a 2 μL water droplet was injected onto the SiO_2_-coated slide immersed in styrene at 25 °C. The equilibrium contact angles were recorded after 60 s using a Young–Laplace fitting. The morphology of all the prepared materials was inspected using FE-SEM on an FEI Sirion 8020 system, which was operated at an accelerating voltage of 5 kV.

The thermogravimetric analysis of the prepared silica and styrene/silica composites was carried out using the TG 209F3 Tarsus thermogravimetric analyzer from NETZSCH Instruments (Germany). The analysis was performed with a heating rate of 10 °C/min, ranging from room temperature to 800 °C, under a nitrogen atmosphere, and the nitrogen flow rate maintained during the process was 100 mL/min.

An ultrasonic cleaning instrument, specifically the KQ-250DE sonicator from Kunshan Ultrasonic Instrument Co., Ltd. (Kunshan, China), was used for pre-emulsification purposes. The surface composition of the silica was comprehensively analyzed using X-ray photoelectron spectroscopy (XPS) on an ESCALAB 250 instrument sourced from Thermo-VG Scientific (Waltham, MA, USA). Moreover, X-ray diffraction (XRD) was carried out to examine the crystal phase of the samples using a TD-3700 model powder diffractometer (Tongda Technology Co., Ltd., Dandong, China).

## 3. Results

### 3.1. Formation of the Polystyrene/Silica Composite

**Figure 2:**
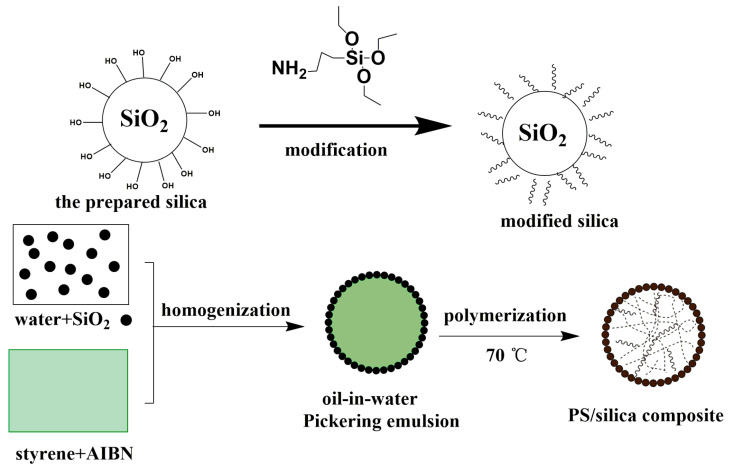
Synthesis strategy for the PS/silica composite obtained by Pickering emulsion polymerization.

**Figure 3:**
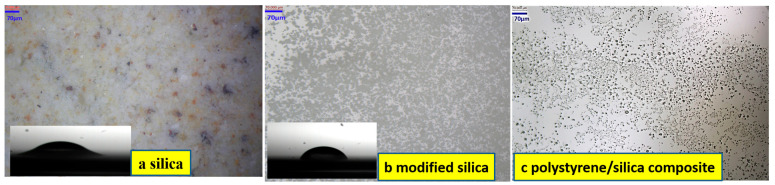
Optical micrographs of the prepared product (**a**) Silica particles; (**b**) Modified silica particles after KH550 treatment; (**c**) Polystyrene/silica composite.

As illustrated in Figure 2, the polymerization of the styrene/silica Pickering emulsions proceeded through interfacial molecular orientation: the hydrophobic alkyl chains were anchored within styrene monomers, while the hydrophilic amino groups extended into the aqueous phase. The AIBN-initiated radical polymerization propagated radially from the silica surfaces, forming covalent Si–O–C linkages at the organic–inorganic interface. The silica derived from the discarded cement exhibited a grayish–white powder morphology. Optical microscopy revealed irregular blocks with certain rough surfaces (Figure 3a). The yellowish tint on the micrograph might be ascribed to the residual iron elements in the waste cement. The surface properties of the synthesized silica were of crucial significance for the stability of the emulsion droplets [18]. To improve these properties, the silane coupling agent KH550 was selected to modify the silica, resulting in the production of modified silica. Microscopic analysis showed that the modified silica particles existed as numerous small spheres, as illustrated in Figure 3b.

Notably, the surface contact angle (θ) is an important parameter for evaluating the wettability of a solid material. Before modification, due to the presence of hydroxyl groups, the silica had a low contact angle of approximately 30°, which indicated strong hydrophilicity, as shown in Figure 3a. After modification, the contact angle increased to 81.9°, as presented in Figure 3b. This increase was caused by the reaction between the KH550 and the silica. During this reaction process, alkyl groups were grafted onto the silica surface, effectively decreasing its hydrophilicity and enhancing its hydrophobicity.

In this research, the modified silica was employed as solid particles for the emulsion preparation. Following the polymerization of the styrene/silica emulsion stabilized by the modified silica, the final product, namely a polystyrene/silica composite, was achieved. Under microscopic examination, numerous small spheres were observed to be embedded in the continuous phase of the composite material, as distinctly shown in Figure 3c. This observation clearly indicated that the synthesized silica had been successfully incorporated into the resin phase.

**Figure 4:**
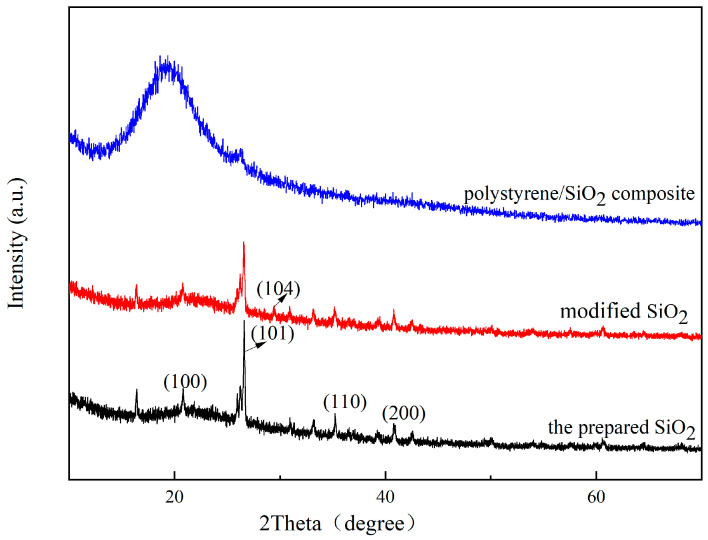
XRD characterizations of the prepared products.

X-ray diffraction (XRD) analysis revealed the structural evolution of the materials (Figure 4). The patterns show the prepared silica, modified silica, and polystyrene/silica composite, with the major diffraction peaks indexed to quartz (SiO_2_, JCPDS No. 12-0708) at 2θ = 20.5° (100), 26.5° (101), 35.3° (110), and 41.0° (200). The characteristic quartz peak at 26.5° persisted in the polystyrene/silica composite but showed asymmetric broadening and intensity reduction, confirming the intact incorporation of SiO_2_ crystallites into the polystyrene matrix [19]. Notably, the modified silica showed a weak impurity peak at 2θ = 29.4° (104), suggesting surface carbonation during the KH550 modification.

**Figure 5:**
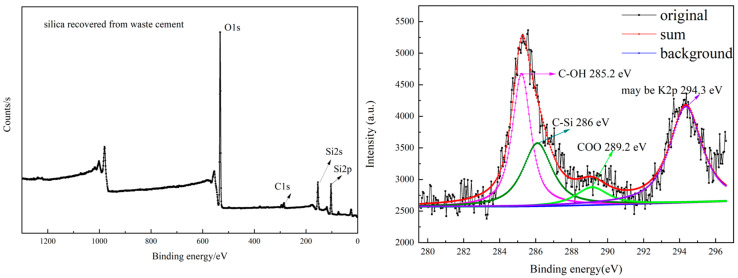
XPS spectra of the prepared silica and its C1s XPS spectra.

**Figure 6:**
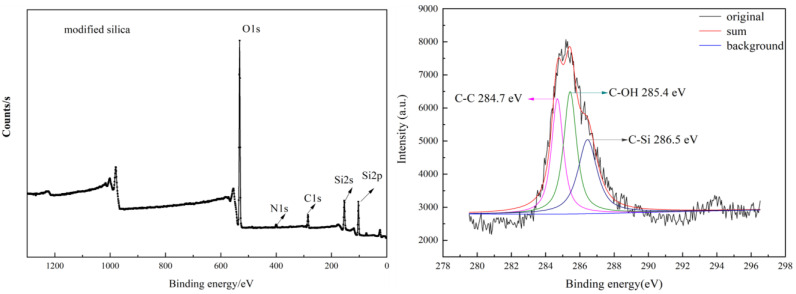
XPS spectra of the modified silica and its C1s XPS spectra.

**Figure 7:**
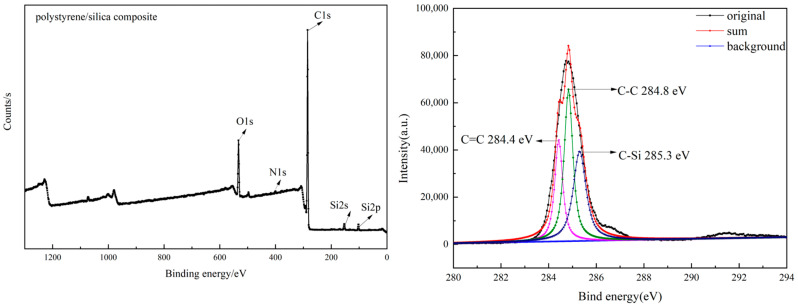
XPS spectra of the PS/silica composite and its C1s XPS spectra.

The prepared products were further evidenced by XPS analysis, as depicted in Figure 5, Figure 6 and Figure 7. Oxygen and silicon elements were obviously detected in the prepared silica, the modified silica, and the polystyrene/silica composite. The prepared silica contained no nitrogen and a very small amount of carbon. However, the content of carbon and nitrogen elements in the modified silica increased significantly compared to that in the prepared silica, indicating the successful grafting of KH550 onto the prepared silica. After polymerization, the carbon content had increased substantially compared to both the prepared and modified silica, while the levels of all the other elements, including silicon, oxygen, and nitrogen, decreased. All of this confirmed the successful polymerization of the Pickering emulsion, resulting in the polystyrene/silica composites. The gradual increase in the carbon content can also be further verified by the C1s XPS spectra. As can be seen on the right in Figure 5, the carbon signal in the silica recovered from cement is very weak, like vibration noise. This weak signal may come from the carbon dioxide adsorbed on the silica surface, including C–OH (~285.2 eV), C–Si (~286 eV), and C(=O) O (~289.2 eV). Moreover, the presence of K2p (~294.3 eV) in the silica recovered from cement serves as compelling evidence of the presence of residual potassium within the silica.

However, the C1s XPS spectra of the modified silica and the polystyrene/silica composite revealed a very strong C signal, which was completely different from that of the unmodified silica. The specific details can be observed on the right of Figure 6 and Figure 7. Additionally, different group structures are present on the right of Figure 6 for the modified silica, namely C–C (~284.7 eV), C–OH (~285.4 eV), and C–Si (~286.5 eV). When the modified silica was used as a stabilizer to prepare the Pickering emulsion and then underwent polymerization, the carbon content of the resulting product increased sharply. Moreover, C–C (~284.8 eV), C = C (~284.4 eV), and C–Si (~285.3 eV) were detected. This indicated that not only was the polystyrene/silica composite successfully obtained but there might also have been residual unpolymerized styrene monomers. The specific changes in the element contents are all listed in Table 2.

### 3.2. Formation of the Pickering Emulsion Stabilized by the Prepared Silica

**Figure 8:**
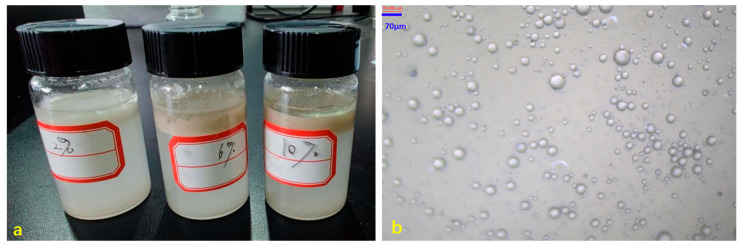
Effect of modified silica concentration on the styrene/water Pickering emulsion: (**a**) Photographs of Pickering emulsions prepared with modified silica at varying concentrations; (**b**) Optical micrograph of the emulsion stabilized by 6 wt% modified silica.

A Pickering emulsion of styrene and water, stabilized by modified silica, was successfully prepared in this study. By using styrene as the oil phase and the modified silica as the stabilizer, an oil-in-water (O/W) Pickering emulsion was achieved [20]. To investigate the impact of the solid particle concentration on the emulsion stability, the amount of modified silica was varied while keeping all the other experimental conditions constant. As shown in Figure 8a, when the modified silica concentration was approximately 2%, the emulsion showed poor stability, with significant styrene separation occurring on the upper layer of the emulsion. However, as the modified silica concentration increased, the delamination phenomenon gradually diminished [21]. Notably, at a particle concentration of 6%, the prepared emulsion exhibited excellent stability, and further increases in the concentration (10%) led to negligible improvements. This behavior could be attributed to the adsorption of silica particles at the oil–water interface [22]. At low modified silica concentrations, there were insufficient particles to fully cover the interface. As a result, oil droplets were able to agglomerate, which hindered the formation of a stable emulsion. Conversely, at high solid particle concentrations (10%), an ample number of particles adhered to the interface, while the excess particles dispersed in the aqueous phase, promoting slight flocculation that effectively stabilized the emulsion [23]. Optical micrographs of the styrene/water Pickering emulsion stabilized by modified silica were also examined. As shown in Figure 8b, many droplets were observed under the optical microscope. It was evident that all these droplets were perfectly spherical, which collectively demonstrated that a Pickering emulsion of styrene and water, stabilized by modified silica, was successfully prepared in this study.

### 3.3. Thermal Stability

**Figure 9:**
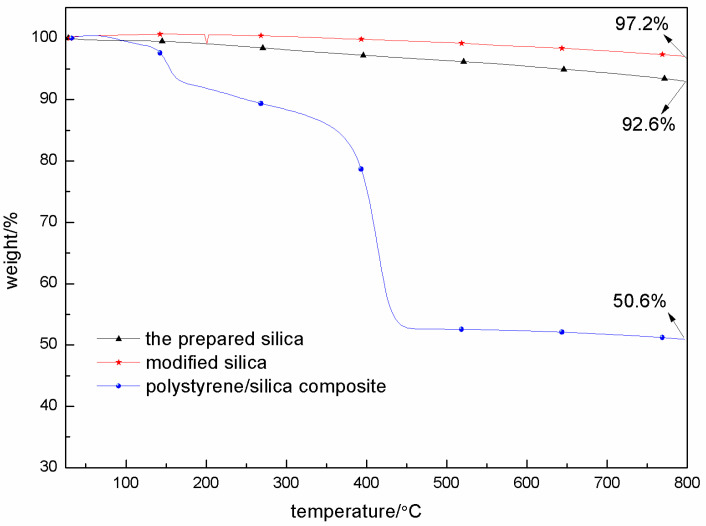
TGA curves of the prepared products.

The thermal stability of the prepared silica, modified silica, and polystyrene/silica composites under a nitrogen atmosphere was investigated using an NETZSCH TG 209F3, as depicted in Figure 9. Evidently, the thermal decomposition rates of these three substances vary significantly. Among them, the modified silica exhibits the lowest thermal decomposition rate, followed by the silica derived from waste cement, while the polymeric Pickering emulsion shows the highest degradation rate. For the cement-derived silica, the 7.4% mass loss at 800 °C is mainly attributed to the surface hydroxyl condensation reaction occurring in the temperature range of 200–800 °C. During this process, as the temperature increases, the surface hydroxyl groups (-OH) on the silica gradually react with each other. The modified silica demonstrates enhanced stability, with only a 2.8% mass loss. This improvement can be ascribed to two main mechanisms. Firstly, the alkyl chains introduced during the modification process shield the reactive -OH groups. These alkyl chains act as a physical barrier, preventing the -OH groups from participating in reactions such as condensation or other chemical reactions that could lead to mass loss, thus reducing the reactivity of the surface. Secondly, the crosslinked siloxane network formed via KH550 plays a crucial role. KH550 contains functional groups that can react with the -OH groups on the silica surface and with each other. Through a series of condensation and polymerization reactions, a three-dimensional crosslinked siloxane network is established. This network structure strengthens the overall framework of the modified silica, making it more resistant to thermal decomposition and contributing to the high carbon yield of 97.2% at 800 °C.

The thermal degradation process of the polymeric Pickering emulsion unfolds in three distinct stages [24]. The primary mass decomposition occurs within the temperature range of 160–440 °C. At 160 °C, the relatively weak intermolecular forces and some labile functional groups in the polymeric structure start to break down, initiating the decomposition process. As the temperature rises, the polymer chains gradually degrade, resulting in the release of volatile products and significant mass loss. When the temperature reaches 440 °C, the residual mass of the polystyrene/silica composite is 53.6%. As the temperature continues to increase, the composite undergoes only minimal further decomposition, maintaining approximately 50.6% of its mass even at 800 °C. This indicates that the incorporation of silica into the polymeric Pickering emulsion significantly enhances the thermal stability of polystyrene [25]. Silica exerts a dual stabilization mechanism. Physically, it acts as a barrier, impeding the diffusion of volatile degradation products. By hindering the escape of these volatile substances, it reduces the rate of further decomposition of the polymer matrix. Chemically, during the thermal degradation process, Si–O–C linkages are formed between the silica and the polystyrene chains. These linkages retard the chain unzipping process, which is a major mechanism for the rapid degradation of polystyrene. By inhibiting chain unzipping, the degradation rate of polystyrene is decreased, thereby enhancing the overall thermal stability of the composite. This finding is highly consistent with the research objective of this paper, which aims to recycle silica from waste cement and utilize it to reinforce the flame retardancy of polystyrene, thus realizing effective waste utilization.

### 3.4. FE-SEM Image Characterization

**Figure 10:**
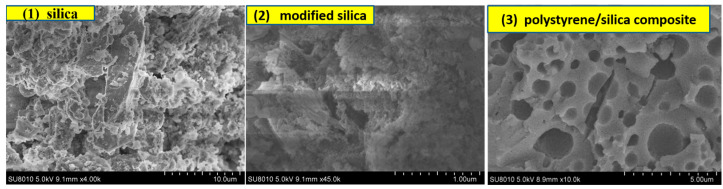
FE-SEM images of the prepared products: (**1**) silica particles recovered from waste cement; (**2**) Modified silica particles after KH550 treatment; (**3**) Polystyrene/silica composite.

To characterize the morphology of the prepared product, the fracture surfaces of the samples were examined using FE-SEM. As illustrated in Figure 10(1), the SiO_2_ recovered from waste cement shows large particles with a rather disordered distribution. After KH550 modification (Figure 10(2)), the modified silica particles display a close packing arrangement. The FE-SEM images of the polystyrene/silica composite in Figure 10(3) reveal that the continuous resin phase is dispersed with many microspheres, which distinctly differ in appearance from the raw silica material derived from waste cement. This visual evidence supports the successful fabrication of polystyrene/silica composite microspheres, which possess an organic–inorganic hybrid structure with a core–shell design [26]. This result is consistent with the findings from the optical micrographs (Figure 3) and XPS chemical bonding analysis (Figure 7).

## 4. Conclusions

This study focused on the recycling and utilization of waste cement, with the aim of fabricating high-performance polystyrene/silica composite materials. Through systematic research and characterization, the major findings are summarized as follows.

Firstly, during the processing of waste cement, silica particles in an initial disordered state with diverse shapes were successfully obtained. The FE-SEM images presented irregular and rugged surfaces. Subsequently, after surface modification with KH550, the silica particles transformed into relatively regular particles. Contact angle analysis confirmed the enhanced amphiphilicity, as the contact angle (θ) increased from 30° to 81.9°, enabling effective stabilization of the Pickering emulsion.

Secondly, a styrene/water Pickering emulsion was successfully prepared by using the modified silica as a stabilizer, and a concentration of 6% was most appropriate. Through the polymerization process, polystyrene/silica composite microspheres were fabricated. FE-SEM characterization clearly confirmed that the resultant composite was composed of numerous spherical microspheres within the continuous resin phase. XPS chemical bonding analysis provided chemical evidence of the interaction between polystyrene and silica.

Thirdly, the TGA results showed that the cement-derived silica exhibited a 7.4% mass loss at 800 °C. After modification, the modified silica demonstrated enhanced stability, with only a 2.8% mass loss at 800 °C. The polystyrene/silica composite retained 50.6% of its mass at 800 °C. The enhanced thermal stability makes this material a promising candidate for applications in high-temperature coatings and flame-retardant packaging composites.

## Figures and Tables

**Table 1:** Formulations of the Pickering emulsions with varying modified SiO_2_ concentrations.

Samples	Modified SiO_2_ (wt%)	Deionized Water (mL)	Styrene (mL)	AIBN (mg)
1	2	15	3	3
2	6	15	3	3
3	10	15	3	3

**Table 2:** Element contents of the prepared silica, modified silica and polystyrene/silica composites.

Samples	Element Analysis on the Surface (wt%)			
	C	Si	O	N
The prepared silica	4.14	26.66	63.59	0
Modified silica	10.97	27.1	59.87	1.91
Polystyrene/silica composites	83.76	3.09	11.72	0.87

## Data Availability

The authors confirm that the data supporting the findings of this study are available within the article.
